# Overactive bladder – 18 years – part I

**DOI:** 10.1590/S1677-5538.IBJU.2015.0365

**Published:** 2016

**Authors:** Jose Carlos Truzzi, Cristiano Mendes Gomes, Carlos A. Bezerra, Ivan Mauricio Plata, Jose Campos, Gustavo Luis Garrido, Fernando G. Almeida, Marcio Augusto Averbeck, Alexandre Fornari, Anibal Salazar, Arturo Dell'Oro, Caio Cintra, Carlos Alberto Ricetto Sacomani, Juan Pablo Tapia, Eduardo Brambila, Emilio Miguel Longo, Flavio Trigo Rocha, Francisco Coutinho, Gabriel Favre, José Antonio Garcia, Juan Castaño, Miguel Reyes, Rodrigo Eugenio Leyton, Ruiter Silva Ferreira, Sergio Duran, Vanda López, Ricardo Reges

**Affiliations:** 1 Escola Paulista de Medicina - EPM - Universidade Federal de São Paulo, SP, Brasil; 2 Departamento de Urologia, Universidade de São Paulo, SP, Brasil; 3 Departamento de Urologia, Faculdade de Medicina do ABC, SP, Brasi; 4 Departamento de Urología, Universidad de los Andes, Bogota, Colombia; 5 Departamento de Urología, Escuela Médico Militar, Cidade do México, Mexico; 6 Cátedra de Urologia, Hospital de Clínicas “José de San Martín”, Buenos Aires, Argentina; 7 Departamento de Urologia, Mãe de Deus Center Hospital, Porto Alegre, RS, Brasil; 8 Universidade Federal de Ciências da Saúde de Porto Alegre, Porto Alegre, RS, Brasil; 9 Departamento de Urologia, AC Camargo Hospital, SP, Brasil; 10 Hospital Clinico de la Fuerza Area de Chile, Santiago, Chile; 11 Instituto Mexicano del Seguro Social, Ciudad de Mexico, Mexico; 12 Departamento de Urologia, Hospital Souza Aguiar, RJ, Brasil; 13 Servicio de Urología, del Complejo Médico Policial Churruca Visca, Buenos Aires, Argentina; 14 Centro Policlínico Valencia “La Viña”, Valencia, Venezuela; 15 Hospital Pablo Tobón Uribe, Medellin, Colômbia; 16 Servicio de Urología, Clinica Indisa, Providencia, Chile; 17 Centro de Reabilitação e Readaptação Dr. Henrique Santillo, Goiânia, Brasil; 18 Servicio de Urología, del Hospital Universitario de Caracas, Caracas, Venezuela; 19 Divisão de Urologia, Universidade Federal do Ceará, CE, Brasil

**Keywords:** Overactive Bladder, Muscarinic Antagonists, Beta-adrenergic agonists, Botulinum Toxin, Sacral neuromodulation, Urodynamics

## Abstract

*Abstract:* Overactive bladder syndrome is one of the lower urinary tract dysfunctions with the highest number of scientific publications over the past two decades. This shows the growing interest in better understanding this syndrome, which gathers symptoms of urinary urgency and increased daytime and nighttime voiding frequency, with or without urinary incontinence and results in a negative impact on the quality of life of approximately one out of six individuals – including both genders and almost all age groups. The possibility of establishing the diagnosis just from clinical data made patients' access to specialized care easier. Physiotherapy resources have been incorporated into the urological daily practice. A number of more selective antimuscarinic drugs with consequent lower adverse event rates were released. Recently, a new class of oral drugs, beta-adrenergic agonists has become part of the armamentarium for Overactive Bladder. Botulinum toxin injections in the bladder and sacral neuromodulation are routine modalities of treatment for refractory cases. During the 1st Latin-American Consultation on Overactive Bladder, a comprehensive review of the literature related to the evolution of the concept, epidemiology, diagnosis, and management was conducted. This text corresponds to the first part of the review *Overactive Bladder 18-years.*

## INTRODUCTION

Overactive bladder syndrome refers to a set of lower urinary tract symptoms that leads to a great negative impact on the quality of life of a significant part of the population. In the last 18 years – since the term “overactive bladder syndrome” was first introduced to the urology community, approximately five thousand articles have been published in specialty journals. During this period, we have witnessed surprising facts, such as the high percentage of people affected by overactive bladder symptoms, against the small number of these affected people seeking medical attention or on adequate treatment. By putting the symptoms of urgency, increased daytime and nighttime voiding frequency and occasional urinary incontinence all together under the same definition, a simplification in establishing the diagnosis and selecting an adequate treatment occurred. Within these almost two decades, a number of drugs have been – and continue to be – added to the arsenal of therapeutic options. Minimally invasive approaches such as the intravesical injection of botulinum toxin and sacral neuromodulation beat off and placed more complex surgical interventions, such as bladder augmentation, as a condition of exception.

Several urological societies proposed guidelines for overactive bladder with the purpose of standardizing its diagnosis and treatment. Although evidence-based medicine provides us with a solid foundation to support the approaches to be adopted, we often see some detachment from what we experience in the daily practice. A variety of systematic reviews have been published on overactive bladder, most of them being focused on specific topics, such as the use of certain drugs, the impact on the quality of life, or even the role of the overactive bladder in selected populations, among which are the elderly and women, or associated with other urinary disorders such as painful bladder syndrome or mixed urinary incontinence.

With the purpose of integrating opinions and experiences of Latin-American urologists, the 1^st^ Latin-American Consultation on *Overactive Bladder* was established. Twenty-seven urologists treating urinary dysfunctions in Argentina, Brazil, Chile, Colombia, Mexico, and Venezuela participated in this event. Five groups were formed, and a topic was assigned to each one to develop: The Concept and Diagnosis of Overactive Bladder; Epidemiology; Conservative Non-Pharmacological Management; Pharmacological Management; and Refractory Overactive Bladder.

A systematic search for publications in the period from 1997 to 2014 was performed in “PubMed” and “Bireme,” which give access to “LILACS,” “IBECS,” “MEDLINE,” “Cochrane Library,” and “SciELO” databases, with the main search term being overactive bladder. The searches, carried out by two independent investigators, targeted each group's assigned topic, as provided in Table 1.

**Table 1:** Method of search of scientific publication at PubMed, Bireme

Groupst	Strategy of search	Filters	Total of identified articles	Total of selected articles
Concept and diagnosis of Overactive bladder	(“Urinary Bladder, Overactive/classification”[Mesh] OR “Urinary Bladder, Overactive/diagnosis”[Mesh] OR “Urinary Bladder, Overactive/etiology”[Mesh] OR “Urinary Bladder, Overactive/pathology”[Mesh] OR “Urinary Bladder, Overactive/physiopathology”[Mesh]) OR (“Urinary Incontinence, Urge/classification”[Mesh] OR “Urinary Incontinence, Urge/diagnosis”[Mesh] OR “Urinary Incontinence, Urge/etiology”[Mesh] OR “Urinary Incontinence, Urge/pathology”[Mesh] OR “Urinary Incontinence, Urge/physiopathology”[Mesh])	English/spanish/portuguese + abstract available + 01/01/1985 a 31/05/2014 + humans + >19 years	799	53
Epidemiology	(Urinary Incontinence, Urge or Urinary Bladder, Overactive) and (economics or epidemiology)	English/spanish/portuguese + abstract available + 01/01/1985 a 31/05/2014 + humans + >19 anos	713	51
Conservative Non-Pharmacological Management	(Urinary Incontinence, Urge or Urinary Bladder, Overactive) and (diet therapy or drug therapy or prevention or control or rehabilitation or surgery or treatment or therapy)	English/spanish/portuguese + abstract available + 01/01/1985 a 31/05/2014 + humans + >19 anos Clinical trials, Review, Randomized Clinical Trial		35
Pharmacological Management			996	71
Refractory Overactive Bladder				26

Initially, 2,508 papers were identified. Incomplete texts, abstracts, and articles on repeated or doubled topics were excluded. The search was limited to articles published in English, Portuguese, and Spanish. The articles forming the database for the present review were selected according to their scientific level of evidence and their relevance for the clinical practice. In the end, 236 articles were selected and forwarded to the members of each group in accordance with the assigned topic. Each group reviewed the selected articles and prepared a summary. The huge number of articles available in the literature made it impossible to include several publications, many of which with unarguable scientific and clinical relevance. When the same topic was addressed in two or more articles, the most recent and complete one was selected.

Each group's summaries were presented in a joint session, and after being discussed and approved, they underwent a compilation and adjustment process that resulted in the present text. The primary objective of this review article is to provide a practical approach about the Overactive Bladder in its different aspects, from concept to treatment of refractory cases according to the interpretation of Latin-American urologists that have great expertise in urinary dysfunctions. The first part will address the overactive bladder concept and classification, epidemiology, diagnosis, and conservative non-pharmacological management.

### Concept and classification of overactive bladder

The term Overactive Bladder was first introduced to the scientific community in 1997 during the Consensus Conference named “*The Overactive Bladder: From Basic Science to Clinical Management*” (1). Since then, the definition of overactive bladder had been reviewed several times and was finally standardized by IUGA (International Urogynecological Association) and ICS (International Continence Society) in 2010 as “urinary urgency, usually with frequency and nocturia, with or without urgency urinary incontinence, in the absence of any urinary tract infection or another obvious pathology” (2). In those terms, therefore, it is a definition based on symptoms only. Although the symptoms are suggestive of detrusor overactivity, this is not a rule of thumb, being more commonly seen in overactive bladder patients with urinary incontinence – also called wet overactive bladder (3). Thus, the terms overactive bladder and detrusor overactivity are distinct, with the latter being a finding in an urodynamic exam. The globally accepted simple and pragmatic definition of overactive bladder meets the purpose of describing a common condition, which allows establishing an initial treatment without having to go through expensive, complex exams. However, there are controversial aspects that could cause confusion, either in the diagnosis or the impossibility of determining how severe the problem is or even in evaluating the treatment results.

The current definition of overactive bladder allows the inclusion of neurological conditions impacting the bladder-sphincter functioning referred as *Neurogenic Overactive Bladder* as well as non-neurogenic conditions – the *Idiopathic Overactive Bladder*. However, in practice, the term *Overactive Bladder* was generally established for non-neurogenic situations. In the present work, we will keep the approach restrict to the *Idiopathic overactive bladder*, simply referred to as *Overactive bladder*.

### Epidemiology of overactive bladder

The prevalence of lower urinary tract symptoms has been studied in several countries, with a methodology that is not always even. Differences in the study population, age, gender, ethnicity, and geographic location, as well as in the inclusion and exclusion criteria impact the results reported by the different studies.

One of the first publications (Milsom et al., 2001) reported a 16.6% prevalence in 16,776 individuals aged 40 years or older, in European countries (4), while the EPIC (**E**uropean Prospective **I**nvestigation into **C**ancer and **N**utrition) study evaluated 19,165 individuals, also in four European countries and Canada, and found a prevalence of 11.8% – with the older the patient, the higher the rates in both genders (5).

A regional study conducted in Austria of an urban population of 2,418, 20-91 year-old men and women showed a 13.5% prevalence, with substantial increase after 40 years of age. Among the European countries, the lowest prevalence was seen in Finland. In a cohort of 3,727 18-79 year-old individuals, the prevalence in that country was only 8%. The authors suggested that the use of inaccurate criteria and population selection may have overestimated the prevalence of overactive bladder reported in other publications (6). In the United States, the NOBLE (**N**ational **O**veractive **B**ladder **E**valuation) study showed a 16.5% prevalence of overactive bladder in a sample of 5,204 adults aged over 18 years (7). In 2010, a multicentric assessment using the OAB-V8 questionnaire was conducted in Venezuela in a total of 3,407 18-75 year-old adults, with 38% of them being males. The prevalence of overactive bladder in this population was 21% (8). Other studies conducted in Latin America found a similar prevalence; 23.4% and 18.9%, in Argentina and Brazil, respectively (9, 10).

Men and women are equally affected. The prevalence of overactive bladder reported by the NOBLE study was 16.9% in women and 16.0% in men (7). In the EPIC study, 13% of women and 11% of men had overactive bladder (5). In Finland, its prevalence in women was 9.3% and 6.5% in men (6). In Venezuela, the study revealed that women were more often affected than men – 25.6% and 13.7%, respectively (8). The same result was observed by Teloken et al., in Brazil, where the condition was reported by 23.3% of women and 14% of men (10), as well as Temml et al., with 16.8% of women and 10.2% of men reporting it (11).

Among the overactive bladder patients, the main reasons to search for medical attention included increased voiding frequency, urinary urgency, and urgency urinary incontinence (12). According to Milsom et al., among the 17% of overactive bladder patients identified in their study, 14% reported increased voiding frequency; 9% reported urgency symptoms; and 6% reported urge-incontinence. About 80% had had the symptoms for at least one year and 49% for at least 3 years (4). Similar data were observed in a multicentric study conducted in Spain involving 1,754 40-92 year-old patients who came to a urological appointment due to their overactive bladder symptoms. The most commonly seen symptoms were urgency (95.2%), nocturia (94.5%), and urinary frequency higher than 8 voids a day (84.2%). In this study, 61% of patients were “unsatisfied” or “very unsatisfied” with their current urinary condition (13).

The impact of untreated overactive bladder is high in the adult population, notably affecting social and professional relationships. All aspects of the quality of life may be impacted, including the psychological, social, household, and sexual function domains. The condition may also reduce one's productivity at work, and in another domain, lead to a repulse behavior towards the sexual activity (7, 14). A British study including men and women showed that 36% believed this disorder “greatly” or “significantly” affected their lives. As a result, major behavioral changes were made by a significant portion of the patients, such as liquid intake reduction by 35%, bathroom mapping by 33%, and going out of home less frequently by 15% (15). Nocturia featuring two or more times per night affects one third of men and women with overactive bladder (5). In the NOBLE study, the quality of life and quality of sleep were significantly impaired among overactive bladder patients (7). A higher risk of falls and fractures among the elderly with urgency urinary incontinence is associated with the rush to reach a bathroom in order to avoid leakages. This age group's higher susceptibility results from the aggravation of the urinary incontinence with ageing together with the diminishing of mobility. Older patients with urgency urinary incontinence are twice as likely to have falls and fractures, when compared with incontinence-free patients (14).

Overactive bladder syndrome negatively impacts the public health system and economic resources as well, when we consider the highest incidence in the older population. It should also be highlighted that the 65+ year-old population will double in the next few decades. The estimated annual cost of overactive bladder in the North-American adult community is as much as 24.9 billion dollars. If we consider those patients occasionally experiencing symptoms too, then the medical and non-medical expenses and indirect costs soar to 36.5 billion dollars (16).

### Diagnosis

The initial evaluation, as it is usual in the medical practice, should include anamnesis and physical exam. During this step, it is critical to determine the onset time of symptoms; their abrupt nature or not; presence of aggravating factors; successful previous treatments; remission periods; need for protection measures against urinary leakages, such as diapers; and the consequent impact of symptoms on the quality of life. Physical exam allows identifying pelvic masses, vesical or intestinal distension, genital prolapse, and increased prostatic volume, which are useful for the differential diagnosis from other pathologies showing a symptomatology similar to overactive bladder. Although we did not include neurogenic conditions in the present review, it is important to perform a brief neurological exam in the context of this evaluation.

Voiding diary is one of the most important tools to assess the lower urinary tract symptoms, as it provides quantifiable, objective data that are also useful to analyze the evolution after the prescription of treatment (17, 18). The voiding frequency and the volume at each void are the main elements of the diary, which allow distinguishing asymptomatic patients and overactive bladder individuals (19–21). In order to verify the trustfulness of the symptoms reported in three-day versus seven-day diaries, Dmochowski et al. studied 881 patients with overactive bladder. Both periods of data collection showed equivalent accuracy and reproducibility, with the three-day diary being considered more convenient by patients (20). Therefore, a three–day voiding diary containing the time and volume of each void and avoiding the inclusion of other information (such as liquid intake) that adds few relevant data and makes patient adherence more difficult is enough to assess the lower urinary tract symptoms in the clinical practice (18).

Questionnaires that evaluate overactive bladder patients are critical measuring tools when conducting scientific studies. They may be didactically classified as specific questionnaires, urinary incontinence questionnaires, quality-of-life questionnaires, and questionnaires of the impact of urinary incontinence on the quality of life. Among the several questionnaires available worldwide, the following show the highest applicability: ICIQ-OAB, OAB-SF, OAB V-8, ICIQ-OABqol (22–25). However, these questionnaires should not be regarded as definitive diagnostic tools. They are designed to standardize and enable data comparison in clinical trials and more objectively measure the impact of the symptoms on patients' quality of life (26–29). It should also be stressed that questionnaires need to be validated in the country language before being applied.

#### Complementary tests

Due to its low cost and non-invasive nature, urinalysis is requested for virtually all patients with overactive bladder symptoms (30, 31). For most patients, once urinary infection and hematuria are ruled out, treatment may be initiated. The presence of microscopic hematuria may suggest the existence of a urinary tract neoplasm or other pathologies, and it deserves additional investigation.

The ultrasound of the urinary tract is not recommended for initial evaluation of overactive bladder patients; however, it may be useful for a differential diagnosis. In addition, it allows measuring the post-voiding residual volume in a non-invasive fashion, and through this, indicating the existence of infravesical obstruction or detrusor failure.

While the diagnosis of overactive bladder is essentially clinical, there is a lot of discussion on the use of urodynamic exam in evaluating the diagnosis of the syndrome. Despite being considered the gold standard for evaluation of low urinary tract symptoms, its invasive nature and the fact that it places a risk of urinary infection usually make the urodynamic exam indicated for refractory patients to the conservative treatments of overactive bladder (32). Another limiting factor to its routine use is the low correlation of urinary symptoms with urodynamic findings in many cases. This was well demonstrated in a review by Digesu, in which the urodynamic results and the clinical diagnosis of overactive bladder matched in 21% of patients only (33). Few guidelines, like the one from France, recommend urodynamic testing for initial evaluation of patients with low urinary tract symptoms (34). On the other hand, the Italian and UK guidelines only recommend the urodynamic investigation in patients at risk of renal complications (35, 36). The urodynamic testing should be performed following good medical practice criteria when symptoms do not allow for a clear diagnosis, when empirical treatment fails, or in cases where more invasive treatments are considered (37, 38).

There is consensus on the fact that performing cystoscopy is not useful to diagnose overactive bladder. Its indication is limited to cases where there is a suspicion of bladder cancer. For example, in the study by Ryan, only 4.1% (14/340) of bladder cancer patients experienced exclusively overactive bladder-related symptoms, with carcinoma in situ being the one with the highest positive correlation (21%) (39).

#### New perspectives for the diagnosis of overactive bladder

The identification of lower urinary tract functional disorder biomarkers, such as for overactive bladder, faces several limitations (17). To date, no biomarkers have demonstrated to be effective as a tool for use in the daily practice. Yet, some bio-markers have been considered useful as potential tools for diagnosis and therapeutic monitoring.

Overactive bladder biomarkers may contribute to the understanding of the urinary storage symptoms. They are involved in the possible overactive bladder-associated changes that occur in the bladder or nervous system and that may be assessed through the dosing of plasma or urine molecules: neurotrophins, adenosine triphosphate (ATP), prostaglandins, C-reactive protein (RCP), and cytokines. Other biomarkers may be assessed through the measurement of bladder wall-related events, such as wall thickness and oxyhemoglobin and deoxyhemoglobin concentration (40).

The neuronal growth factor (NGF) modulates the release of neurotransmitters and reduces the sensitivity threshold of nociceptive fibers; therefore, its neutralization diminishes the hyperreflexia in an animal model of spine injury (41). The NGF is produced in the urothelium and vesical smooth musculature. Clinical and experimental data have shown a direct association between the increased NGF expression in the vesical tissue and in the urine (42–44). However, an issue in the use of NGF as an overactive bladder biomarker is the fact that it can be elevated in other clinical conditions as well, such as painful bladder syndrome, urinary infections, urinary lithiasis, and bladder tumors (45, 46). Qu et al. conducted a meta-analysis that identified only eight controlled studies (47). In all of them, patients with overactive bladder symptoms showed higher urinary NGF levels than healthy individuals and achieved lower NGF levels after having been effectively treated. In the subgroup analysis, the NGF was demonstrated to be higher in wet overactive bladder than dry overactive bladder patients. However, no differences were found when the NGF levels of overactive bladder patients and painful bladder syndrome patients were compared. The authors concluded that the urinary NGF could be a useful biomarker to diagnose overactive bladder and a potential biomarker to differentiate cases with and without associated urinary incontinence, but it could not be used to differentiate overactive bladder from painful bladder syndrome (46).

Urinary prostaglandins and cytokines were also proposed as overactive bladder markers. Prostaglandin E2 (PGE2) was more widely studied and has been considered as superior to NGF as a discriminatory marker. According to the study by Kim et al., the PGE2 levels were lower in a subgroup of overactive bladder and detrusor hypocontractility patients – as demonstrated in the urodynamic testing – than in overactive bladder patients without detrusor hypocontractility (44). Other cytokines were also high in the urine of overactive bladder patients, including monocyte chemotactic protein-1 (MCP-1) and the soluble fraction of the CD40 ligand (scd40L) (48, 49). However, their exclusive association with overactive bladder symptoms is not well established. This was also seen with the dosing of other biomarkers such as RCP and ATP with low sensitivity and specificity (50–55).

### Non-pharmacological and non-surgical management of overactive bladder

It is important that patients understand that overactive bladder is not a disease; rather, it is a syndromic complex and it is not life-threatening. After evaluation and rule-out of other conditions that could require further investigation, patients should be educated about the therapeutic options, including the non-treatment.

Non-pharmacological conservative treatment of overactive bladder may involve a number of measures targeting to increase the voiding interval, improve bladder capacity, reduce urinary urgency and nocturia episodes, and prevent involuntary loss of urine. Interventions in lifestyle-related factors, collectively referred to as behavioral therapy, were associated with potential improvement of symptoms. However, there are few studies with high level of scientific evidence evaluating specific chances in the lifestyle as the single treatment.

The majority of the studies evaluating the influence of caffeinated beverage consumption indicate that this may worsen the overactive bladder symptoms and urinary incontinence, due to either their diuretic proprieties or their potential bladder irritation effect (56–59). Bryant et al., in a randomized clinical trial, compared women with a daily consumption of 965 mg of caffeine and a control group with an average consumption of 279 mg (60). In the control group, lower urgency (61% vs. 12%) and fewer episodes of incontinence (55% vs. 26%) was observed, although the values did not reach statistical significance. In another randomized, crossover, double-blind study, though, the replacement of caffeine by decaffeinated beverages did not show to improve the patients' urgency and frequency symptoms (61).

Swithinbank et al. evaluated the effect of liquid intake reduction in 30 women with idiopathic detrusor overactivity and observed a significant reduction of voiding frequency, urgency and incontinence symptoms as well as improvement of the quality of life (62). In a controlled study of overactive bladder patients, a significant reduction of frequency, urgency and nocturia was observed when patients reduced the liquid intake by 25% or more (63). However, restricting the consumption of liquids may have adverse effects, including urinary infection predisposition, dehydration, calculus formation, and constipation, which contribute to the development of an overactive bladder (64).

Bladder training is a behavioral intervention that has the purpose of progressively increasing the voiding intervals. It tries to restore the normal micturition function by scheduling voids by the clock in order to increase the intervals between them. Fantl et al., in a randomized clinical trial of bladder training compared with a group with no intervention, showed a beneficial effect of the former on the overactive bladder symptoms, with a 57% reduction of urinary incontinence episodes (65). This study's weakness, however, was the small number of patients with exclusively overactive bladder. Burgio et al., in another randomized study of 307 women with urgency urinary incontinence, compared the use of an oral anticholinergic drug, behavioral therapy, and both treatments together (66). Both therapeutic groups showed improvement of urgency, without any significant difference. Even though, in the group treated with oral medication plus behavioral therapy, the voiding frequency significantly improved (66). A systematic meta-analysis of Cochrane Library conducted by Rai et al. suggested that patients are more likely to have favorable results when they are treated with oral anticholinergics alone than when undergoing bladder training techniques alone (67). Although the meta-analysis favored the anticholinergic-treated group, it is important to stress that no individual study showed a statistically significant difference. In this study, also the treatment combination (oral anticholinergic + bladder training) was superior to the bladder training as a monotherapy (67).

Historically, pelvic floor physiotherapy has been used in the treatment of stress urinary incontinence (68). However, the contraction of pelvic floor muscles may have an inhibitory effect on the detrusor contraction. For this treatment modality, biofeedback and external or intracavity electrostimulation may be used as additional resources. Kafri et al., in a randomized clinical trial, divided urgency urinary incontinence patients into four groups: treatment with anticholinergic drugs, bladder training, pelvic floor physiotherapy, and a combination of the three treatments (69). In this study, a significant improvement was observed in all treatment groups, but those receiving the treatment combination experienced a significant voiding frequency reduction in more than one-year follow-up (69).

One of the limitations of behavioral therapy and pelvic floor exercises is the patient's adherence to treatment. Studies showed that up to 81% of patients adhered to the initial recommendations; 12 months later, however, less than one third of patients were continuing the exercises. Most women tend to adhere to the exercises during supervised intervention, but their participation diminishes over time (70). Supervised intervention can be carried out in group in order to reduce costs. The results from individual sessions and group sessions were similar, although patients expressed their preference to individual training (71).

Some studies compared different modalities of electrical stimulation and oral medication. Franzen et al. randomized a group of patients for treatment with tolterodine or for vaginal or anal electrical stimulation (72). A significant reduction of the voiding frequency was observed within 24 hours in both groups. At 6 months, 73% of patients receiving electrical stimulation and 71% of patients treated with oral tolterodine reported less discomfort due to the bladder symptoms and similar improvement of the quality of life, as measured with the King's Health Questionnaire (72). Another randomized study conducted by Ozdeleli et al. compared transvaginal electrical stimulation with oral trospium by using urodynamic parameters and quality-of-life scales in 35 women (73). Both treatments showed similar improvement of symptoms. The discontinuation of treatment, however, caused deterioration in most of the objective and subjective overactive bladder symptoms.

Berghmans et al. conducted a systematic review and meta-analysis of the electrical stimulation in males that revealed the existence of only one study in which the real stimulation was more effective than the fictitious one, despite losing effect within up to six months (74). In all four studies included, there was no compelling evidence of improvement from the association of electrical stimulation with pelvic floor exercises when compared with the exercises as monotherapy.

In 2012, Farag et al. reviewed 17 articles, 15 of which included superficial percutaneous nerve stimulation of the clitoris (75). An increase of 11-144% of the bladder capacity was observed in 14 of the studies. Goldman et al., in a prospective, observational, multicentric study reported a reduction of at least half of the leaking episodes (pad test) in 76% of women (76). In the review of the Cochrane Library, different types of electrical stimulation, such as transcutaneous, percutaneous and intracavity was observed to show healing rates similar to the pharmacological therapy. Improvement rates tend to favor the electrical stimulation. However, these data were only significant for one clinical trial, and confirming them with further studies in the future is recommended (67). An alternative neuromodulation therapy may be used for transcutaneous electromagnetic stimulation of sacral roots. O'Reilly et al., in a randomized, double-blind clinical trial involving 63 patients undergoing trans-sacral magnetic stimulation in comparison to a sham group could not demonstrate any significant difference regarding the increase of the maximum voided volume in the pad test and the visual analogic scale. Neither could they find a difference in the quality-of-life scale (77).
